# A protocol for vaginal wall-derived organoids from women with pelvic organ prolapse and benign controls: limited applicability to pathological uterosacral ligament tissues

**DOI:** 10.3389/fcell.2026.1977193

**Published:** 2026-10-23

**Authors:** Xia Yu, Sihan Wu, Qin Tao, Xinyu Luo, Yanfeng Huang, Xiu Yang, Xiao Yang, Yonghong Lin

**Affiliations:** 1 Department of Clinical Laboratory, Chengdu Women’s and Children’s Central Hospital, School of Medicine, University of Electronic Science and Technology of China, Chengdu, Sichuan, China; 2 Department of Obstetrics and Gynecology, Chengdu Women’s and Children’s Central Hospital, School of Medicine, University of Electronic Science and Technology of China, Chengdu, Sichuan, China; 3 Hunter Biotechnology, Inc., Hangzhou, Zhejiang, China; 4 Department of Operating Room, Chengdu Women’s and Children’s Central Hospital, School of Medicine, University of Electronic Science and Technology of China, Chengdu, Sichuan, China

**Keywords:** epithelial cells, organoids, pelvic organ prolapse, uterosacral ligament, vaginal wall

## Abstract

**Purpose:**

Pelvic organ prolapse (POP) significantly impairs women’s quality of life; however, the lack of reliable *in vitro* human-derived models has hindered the elucidation of its pathogenesis and the development of targeted therapies. This study aims to establish a reproducible protocol for generating patient-derived organoids from human vaginal wall and uterosacral ligament (USL) tissues and to define the boundaries of applicability of this model for POP research.

**Methods:**

Vaginal wall and USL tissues were obtained from patients with POP (stage ≥ III, n = 3) and benign controls (n = 2). Each biological sample was tested in three independent technical replicates for two parallel culture approaches, including dissociated primary cells and digested tissue fragments. The tissues were processed and digested using a combination of collagenase II and DNase. Primary cells and digested tissue fragments were cultured in a three-dimensional (3D) Matrigel system with specialized organoid culture medium supplemented with growth factors, including Wnt3a, R-Spondin 1, and Noggin. Successful organoid formation was prospectively defined as the formation of discrete, three-dimensional spherical or cystic structures with a clearly visible border, sustained growth for at least 7 days under bright-field microscopy, and a diameter ≥100 µm. Organoid formation, growth, and morphology were monitored over time. Immunofluorescence staining was performed on the organoids generated from patients with POP to analyze their cell composition. KRT14 (epithelial), vimentin (fibroblast-related), α-SMA (myofibroblast/smooth muscle-related), VWF (endothelial), and SM22 (smooth muscle) were detected.

**Results:**

Under light microscopy, primary cells from benign control vaginal wall tissue formed organoids within the first week of culture. By Day 11, clear three-dimensional structures measuring approximately 100–500 μm in diameter were observed. In contrast, vaginal wall-derived organoids from patients with POP exhibited similar morphology but were fewer and less dense. When undigested tissue fragments were cultured for 48 h to form cell aggregates and then dispersed and reembedded in Matrigel, the organoid yield was significantly greater than that when the cultures were initiated with discrete primary cells alone, and organoid growth was maintained through the second passage. Both primary cells and tissue fragments from benign control USL generated organoids, whereas USL tissues from patients with POP did not form organoids under identical conditions. Immunofluorescence analysis of vaginal wall-derived organoids revealed expression of the epithelial marker KRT14, whereas vimentin, α-SMA, VWF, and SM22 were not detected. These findings indicate that these organoids were predominantly composed of epithelial cells.

**Conclusion:**

We present a preliminary protocol for generating vaginal wall-derived organoids from both patients with POP and benign controls, which may serve as a useful *in vitro* tool for studying epithelial biology in POP. Importantly, pathological USL tissues from patients with POP failed to form organoids under identical conditions, whereas benign control USL did; this negative result defines a current applicability boundary and indicates that the protocol is not yet suitable for generating organoids from pathological USL or for modeling USL-derived pathology. Further optimization and validation in larger cohorts are therefore needed before POP research can be broadly applied.

## Introduction

Pelvic organ prolapse (POP) is a prevalent gynecological condition characterized by the descent of the anterior or posterior vaginal wall, the uterus or the vaginal apex, often resulting in bothersome symptoms, including pelvic pressure, voiding dysfunction, and defecatory difficulties that severely compromise women’s quality of life (Vergeldt et al., 2015). Epidemiological projections estimate that the global incidence of POP will reach 156 million by 2036, posing substantial socioeconomic burdens on healthcare systems worldwide (Li P. et al., 2025). Despite its high prevalence, the etiological mechanisms of POP remain inadequately understood. Surgical intervention remains the mainstay of treatment, yet recurrence rates are high, with up to 30% of patients requiring repeat surgery within 5 years (Poutakidis et al., 2025). This therapeutic plateau largely reflects our limited understanding of the cellular and molecular events that govern pelvic floor support.

The study of POP pathophysiology has been hindered by the lack of reliable experimental models. Animal models, while informative, fail to recapitulate the unique biomechanical and anatomical features of the human pelvic floor (Li et al., 2026). Conventional two-dimensional (2D) cell cultures derived from patient tissues allow basic mechanistic studies but rapidly lose tissue-specific phenotypes and cellular heterogeneity on passaging (van Velthoven et al., 2024). Furthermore, tissue biopsies provide only static snapshots, precluding longitudinal investigations of disease progression or therapeutic responses (Gong and Xia, 2019). These limitations underscore the urgent need for physiologically relevant, patient-derived *in vitro* models that preserve the cellular architecture and functional characteristics of native pelvic floor tissues.

Organoid technology has emerged as a powerful platform for modeling human diseases and advancing personalized medicine (Xu et al., 2026; Bhattacharya et al., 2025). By leveraging the self-renewal and differentiation capacities of tissue-resident adult stem cells, organoids recapitulate key structural and functional features of their organs of origin in a three-dimensional (3D) microenvironment. This approach has been successfully applied to study a wide range of tissues, including the intestine, brain, and endometrium (Okamoto et al., 2025; Ciprietti et al., 2025). In gynecological research, organoids derived from endometrial, placental, and ovarian tissues have provided novel insights into implantation, trophoblast invasion, and ovarian carcinogenesis (Li Y. et al., 2025; Gnecco et al., 2023). However, the application of organoid technology to pelvic floor disorders, particularly POP, remains underexplored.

In this study, we aimed to establish a reproducible protocol for generating organoids from two key pelvic floor tissues: the vaginal wall and the uterosacral ligament (USL). Our protocol was designed to systematically evaluate organoid generation from both dissociated primary cells and digested tissue fragments (Figure 1), using a Wnt3a/R-Spondin 1/Noggin-based culture formulation optimized for epithelial stem cell expansion. We further characterized the cellular composition of the resulting organoids, which formed in cultures from both patients with POP, using immunofluorescence staining for KRT14 (epithelial), vimentin (fibroblast-associated), α-smooth muscle actin (α-SMA) (myofibroblast/smooth muscle-associated), von Willebrand factor (VWF) (endothelial), and smooth muscle 22 alpha (SM22) (smooth muscle). By providing a detailed, step-by-step protocol with comprehensive troubleshooting, this work aims to fill a critical methodological gap and offer the research community a standardized platform for investigating POP pathophysiology and evaluating potential therapeutic interventions.

**FIGURE 1:**
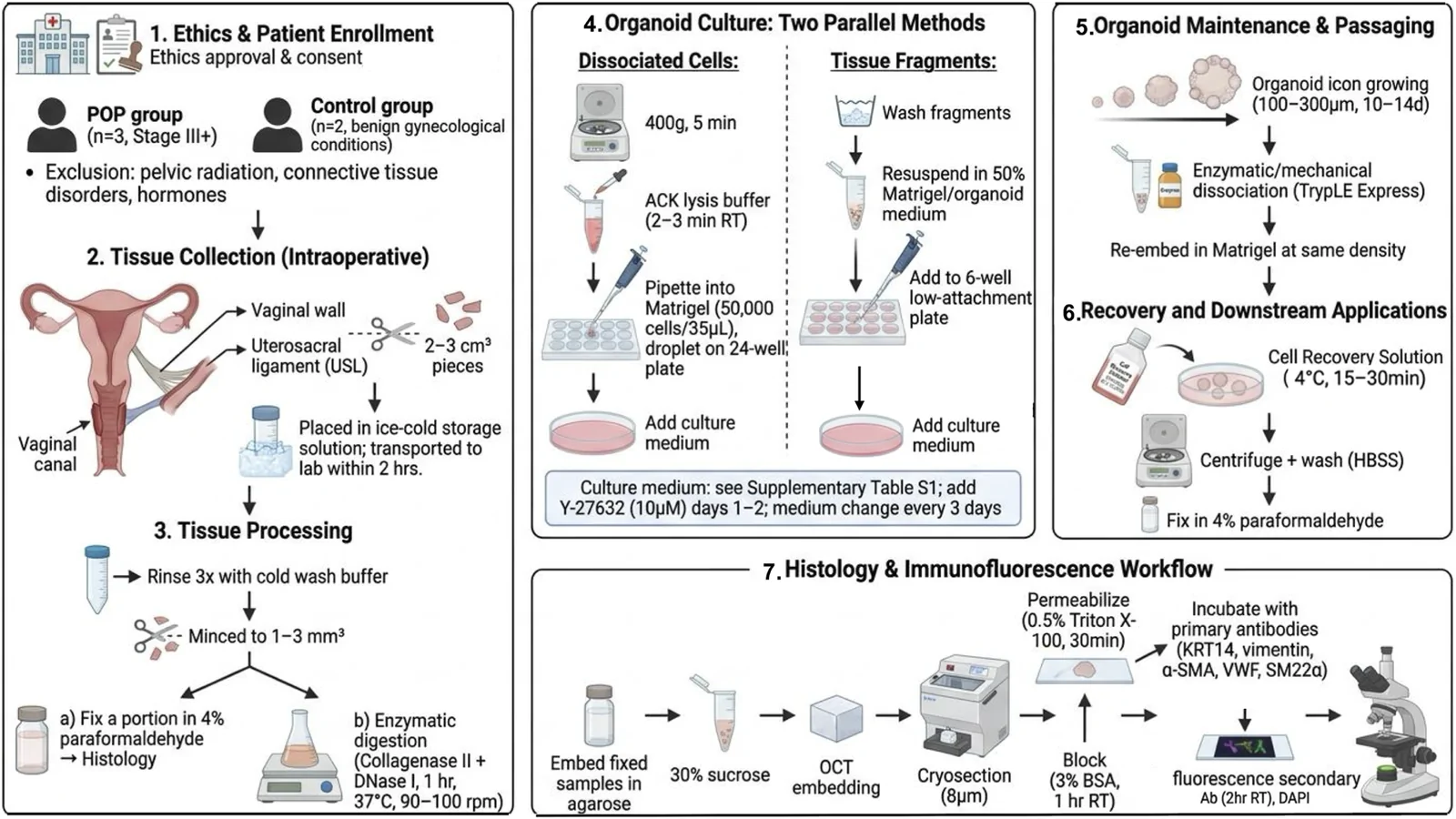
Flowchart of the method for constructing organoids from vaginal well and uterosacral ligament tissues.

## Materials and methods

### Study design and patient enrollment

This study was approved by the Ethics Committee of Chengdu Women’s and Children’s Central Hospital [Approval No. 2022 (131)] and conducted in accordance with the Declaration of Helsinki. Written informed consent was obtained from all participants before surgery.

Patients who underwent hysterectomy at our institution between April 2024 and October 2024 were recruited. The POP group included three patients with stage III or higher POP according to the POP Quantification (POP-Q) system who underwent total hysterectomy with concurrent partial vaginectomy when clinically indicated. The control group included two patients who underwent total hysterectomy for benign gynecological conditions (uterine fibroids), none of which were known to significantly affect vaginal or ligamentous tissues. All the patients had no history of pelvic radiation, connective tissue disorders, or hormonal therapy within 6 months before surgery.

### Tissue Collection and Processing

Vaginal wall tissue and USL samples were collected intraoperatively. Immediately after excision, the tissues were rinsed with sterile saline to remove blood debris, cut into approximately 2–3 cm^3^ pieces, and submerged in ice-cold tissue storage solution (Supplementary Table S1). The samples were transported to the laboratory on ice and processed within 2 h of collection to maximize cell viability.

On arrival, the tissues were washed three times with precooled wash solution (Supplementary Table S1) and mechanically minced into 1–3 mm^3^ fragments using sterile surgical scissors. A portion of the tissue was fixed in 4% paraformaldehyde for histological analysis. The remaining tissue fragments were subjected to enzymatic digestion in a solution containing 2.5 mg/mL collagenase II and 0.5 mg/mL DNase I in DMEM with moderate agitation (90–100 rpm) at 37 °C for 1 h. Digestion was stopped by the addition of DMEM supplemented with 10% fetal bovine serum (FBS). The resulting cell suspension was filtered through a 100-μm cell strainer to separate dissociated primary cells from undigested tissue fragments.

### Organoid culture conditions

Organoids were generated using two parallel approaches, with three independent technical replicates per approach for each biological sample: (1) dissociated primary cells and (2) digested tissue fragments retained on the cell strainer. Detailed step-by-step procedures are provided in the Supplementary Methods, and the troubleshooting steps and solutions are listed in Supplementary Table S2.

For the dissociated primary cell approach, the cells were pelleted by centrifugation (400 g, 5 min), treated with ACK lysis buffer (2–3 min at room temperature) to remove erythrocytes, and their viability was assessed by trypan blue exclusion before plating. Only samples with >50% viable cells were used. Approximately 50,000 viable cells were resuspended in Matrigel (Corning, #356231) and plated as 35-µL droplets onto prewarmed 24-well plates. After solidification at 37 °C for 20 min, 700 µL of organoid culture medium was gently added to each well.

For the tissue-fragment approach, fragments retained on the 100-µm cell strainer after digestion were collected, washed, and resuspended in 3 mL of organoid culture medium containing 50% Matrigel. The suspension was then transferred to 6-well low-attachment plates to promote aggregate formation. Fragment input was not quantified by cell number because of the heterogeneous size of the retained fragments.

Both conditions were derived from the same initial tissue sample (2–3 cm^3^ per sample) and subjected to the same digestion procedure. The organoid culture medium is described in Supplementary Table S1. For the first 2 days of culture, 10 µM Y-27632 (a ROCK inhibitor) was added to the medium to prevent anoikis. Afterward, the medium was replaced every 3 days.

### Organoid passaging and recovery

Successful organoid formation was prospectively defined as the appearance of discrete, three-dimensional, spherical or cystic structures with a clearly visible border, sustained growth for at least 7 days under bright-field microscopy, and a diameter ≥100 µm. Loose cell aggregates, single cells, and undigested tissue fragments were not counted as organoids. Organoids were passaged when they reached 100–300 μm in diameter, approximately every 10–14 days. For passaging, the organoids were mechanically dissociated and enzymatically digested with TrypLE Express at 37 °C for up to 10 min, with intermittent pipetting to achieve small cell clumps (5–20 cells per clump). Digestion was stopped with DMEM containing 2% FBS, and the dissociated cells were reembedded in Matrigel at the same density as described above.

For downstream applications, the organoids were recovered from the Matrigel using Cell Recovery Solution (Corning, #354253) at 4 °C for 15–30 min with gentle agitation, followed by centrifugation (400 g, 5 min) and washing with precooled HBSS. Recovered organoids were fixed in 4% paraformaldehyde for 2 h at room temperature and processed for histological or immunofluorescence analysis.

### Immunofluorescence staining

Organoids derived from patients with POP and their matched tissue samples were fixed and embedded in agarose and dehydrated in 30% sucrose solution at 4 °C overnight, followed by OCT embedding and cryosectioning at 8 μm thickness. HE staining and immunofluorescence staining were carried out. For immunofluorescence staining, the sections were permeabilized with 0.5% Triton X-100 for 30 min, blocked with 3% bovine serum albumin (BSA) for 1 h at room temperature, and incubated overnight at 4 °C with primary antibodies against KRT14 (1:500), vimentin (1:500), α-smooth muscle actin (α-SMA (1:100), von Willebrand factor (VWF (1:100), and smooth muscle 22 alpha (SM22 (1:200). After thorough washing, the sections were incubated with appropriate Alexa Fluor-conjugated secondary antibodies for 2 h at room temperature, counterstained with DAPI, and mounted with antifade mounting medium. Images were acquired using a fluorescence microscope (Olympus, Japan).

## Results

### Organoid generation from vaginal wall tissues

After primary cell plating, organoids derived from benign control vaginal wall tissues (CON-CY) were consistently observed within the first week and progressively enlarged from Day Day 11 (Figure 2A). By Day 11, well-defined, three-dimensional structures were clearly visible, with sizes ranging from approximately 100 μm–500 µm under higher magnification.

**FIGURE 2:**
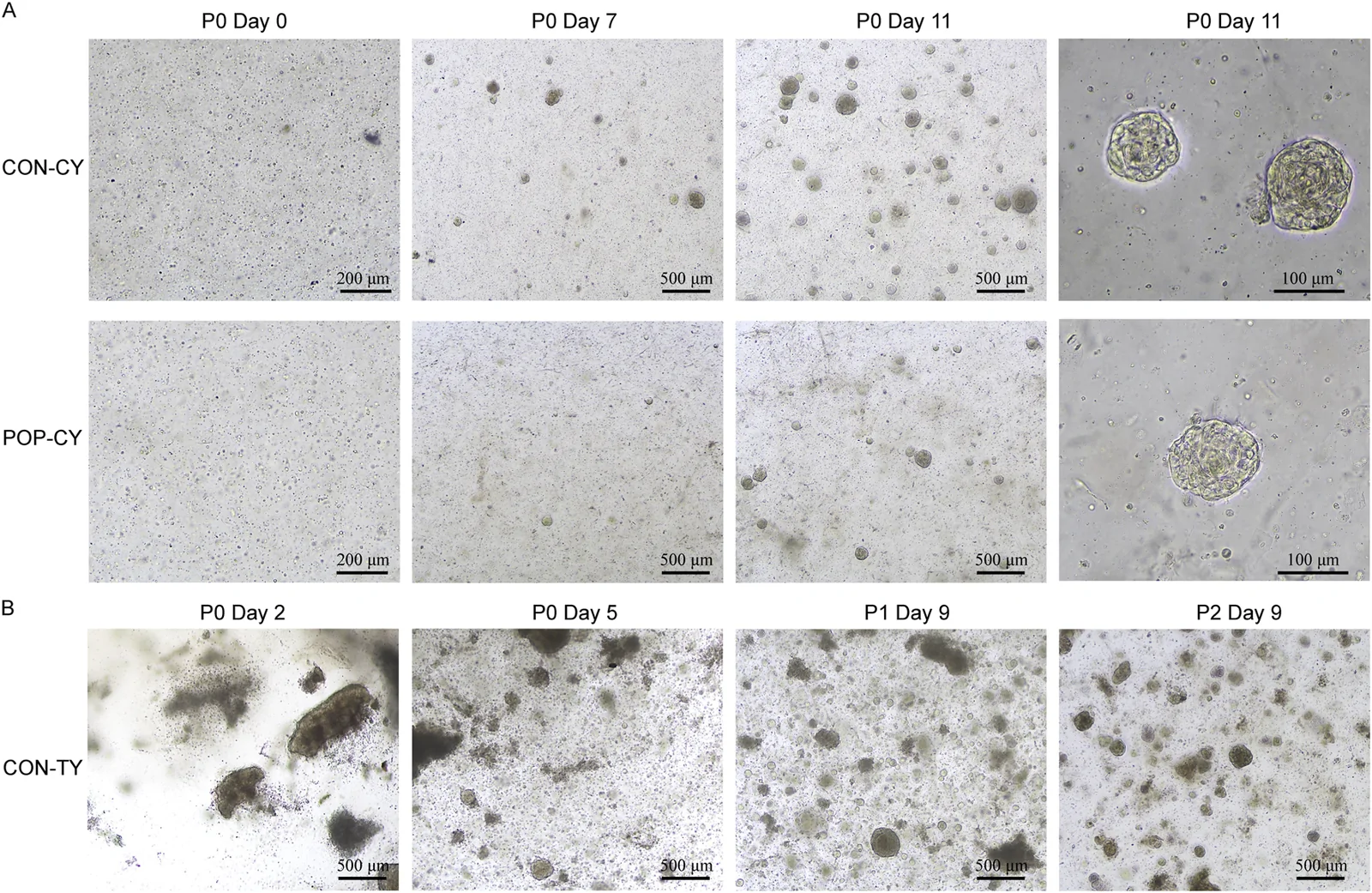
Generation of organoids from vaginal wall tissues. **(A)** Primary cells derived from benign control vaginal tissue (CON-CY, n = 2) and from the vaginal tissue of patients with pelvic organ prolapse (POP-CY, n = 3) were cultured in organoid-forming medium. Representative bright-field images at P0 Day 0, P0 Day 7, and P0 Day 11 are shown. The rightmost panels show higher-magnification views of the organoids formed in the CON-CY and POP-CY cultures at P0 Day 11. Organoids formed in both the CON-CY and POP-CY cultures. **(B)** Digested tissue fragments from benign control vaginal tissue (CON-TY, n = 2) were cultured in organoid-forming medium. Representative images at P0 Day 2, P0 Day 5, P1 Day 9, and P2 Day 9 show progressive organoid formation and serial passaging. Scale bars: 200 µm (A, left), 500 µm (A, middle two panels and B), and 100 µm (A, right). P0, P1, and P2 indicate passage numbers; day indicates the day of culture; and N indicates the number of biological samples. For each biological sample, three independent technical replicates were performed.

In contrast, the growth patterns of the organoids generated from vaginal wall tissues from patients with POP markedly differed (Figure 2A). Despite their morphological resemblance to healthy organoids under the microscope, they exhibited lower formation efficiency. At the corresponding time points (Day 7 and Day 11), the number of POP-derived organoids was clearly lower than that of the healthy controls, and the overall density of organoid structures per visual field was reduced. However, because the sample size was small (three patients with POP and two controls) and no quantitative counting or statistical analysis was performed, these observations are qualitative and preliminary. These findings suggest that pathological vaginal tissues from patients with POP may possess either a reduced stem/progenitor cell pool or impaired self-organization capacity under the same culture conditions.

Importantly, when undigested tissue fragments retained on the filter were cultured separately, we observed that in treated tissues (CON-TY) mixed with Matrigel and plated in low-adhesion plates, some tissue cells coalesced to form compact cell aggregates after 48 h of culture. After these aggregates were dissociated and reembedded in Matrigel, microscopic examination revealed that compared with parallel cultures initiated solely from dissociated primary cells, they gave rise to a greater number of organoids (Figure 2B). In CON-TY, organoid formation was observed on Day 5 after plating (P1 Day 5), and with continued culture to Day 9 (P1 Day 9), most of the organoids increased in volume, suggesting a growth trend during this process. After another round of dissociation and passaging, organoid growth was still observed on Day 9 after replating (P2 Day 9). In the present study, organoid growth was observed up to passage 2. Long-term self-renewal and sustained expandability beyond this passage level were not established and require further demonstration. Moreover, because the sample size was small (POP n = 3; benign controls n = 2; three independent technical replicates per biological sample) and no quantitative counting or statistical analysis was performed, these observations are qualitative and preliminary.

### Organoid generation from uterosacral ligament tissues

Furthermore, given the established pathological relevance of uterosacral ligaments in POP, we extended our organoid generation approach to tissues derived from this anatomical site. As illustrated in Figure 3A, primary cells (CON-CD) and tissue fragments (CON-TD) from normal uterosacral ligaments successfully gave rise to solid, three-dimensional organoids, confirming that this tissue type also contains organoid-competent cell populations.

**FIGURE 3:**
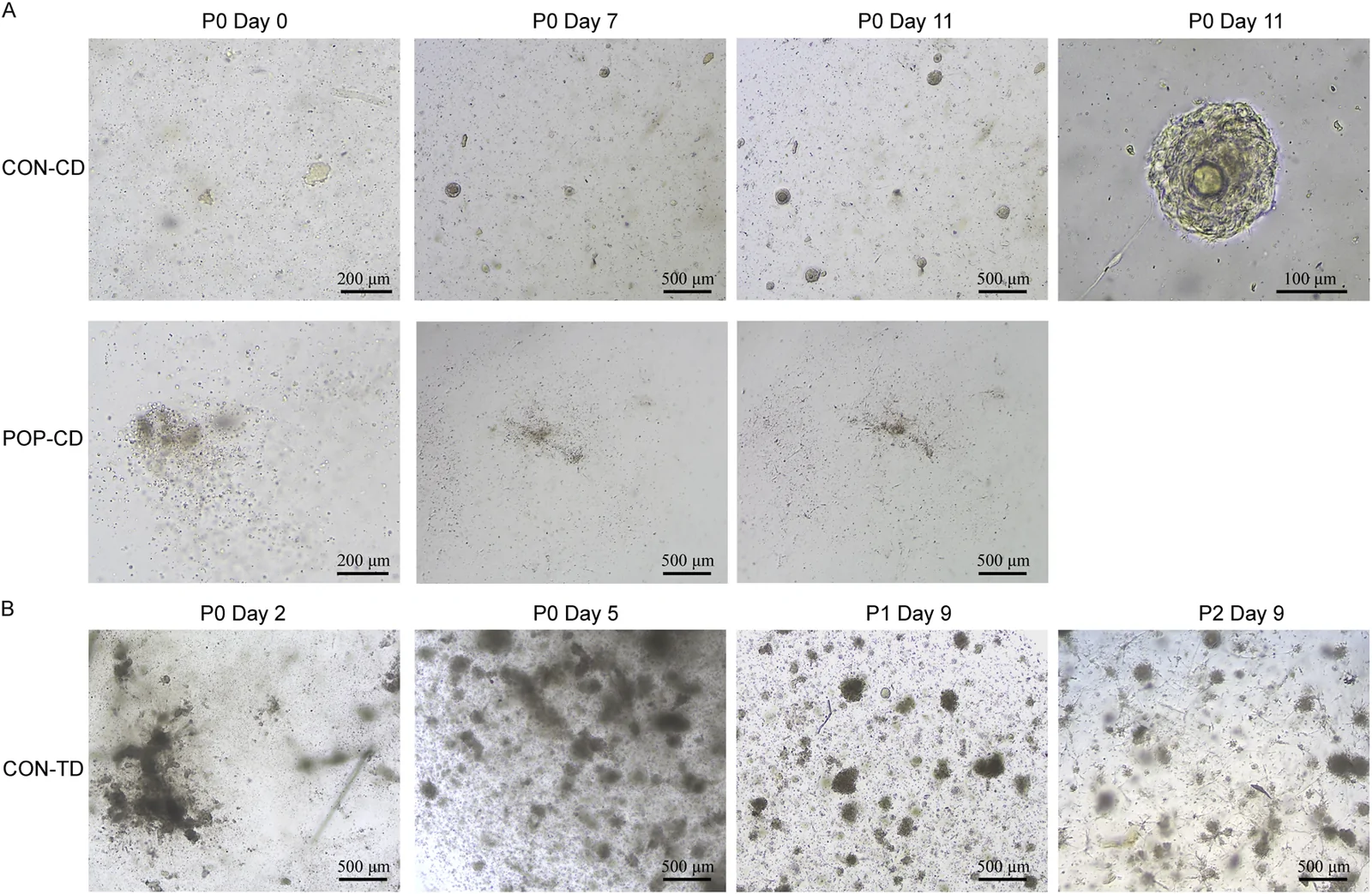
Generation of organoids from uterosacral ligament tissue. **(A)** Primary cells derived from benign control uterosacral ligaments (CON-CD, n = 2) and from the uterosacral ligaments of patients with pelvic organ prolapse (POP-CD, n = 3) were cultured under organoid-forming conditions. Representative bright-field images at P0 Day 0, P0 Day 7, and P0 Day 11 are shown. The rightmost panel shows a higher-magnification view of an organoid formed in the CON-CD group at P0 Day 11. Organoids were observed in the CON-CD cultures, whereas the POP-CD cultures produced only cell aggregates, not organoids. **(B)** Digested tissue fragments from benign control uterosacral ligaments (CON-TD, n = 2) were cultured under organoid-forming conditions. Representative images at P0 Day 2, P0 Day 5, P1 Day 9, and P2 Day 9 show progressive organoid formation and passaging. Cultures from digested tissue fragments of patients with POP did not yield organoids and are therefore not shown. Scale bars: 200 µm (A, left), 500 µm (A, middle two panels and B), and 100 µm (A, right). P0, P1, and P2 indicate passage numbers; day indicates the day of culture; and N indicates the number of biological samples. For each biological sample, three independent technical replicates were performed.

In marked contrast, despite the use of the same culture conditions and parallel strategies, both primary cell plating (POP-CD) and undigested fragment culture (POP-TD), no organoid formation was observed in pathological uterosacral ligament tissues from patients with POP (Figures 3A,B).

### Immunofluorescence characterization of vaginal wall-derived organoids from patients with POP

Immunofluorescence staining was performed only on organoids derived from the vaginal wall that were successfully cultured from patients with POP. For the organoids derived from USL, which were successfully cultured only from the benign control, immunofluorescence staining was not detected. Before immunofluorescence staining, HE staining was first performed on the generated organoids and matched tissues to evaluate their overall morphology, structural organization, and cellular composition and to confirm their epithelial characteristics. As shown in Figure 4A, the matched vaginal wall tissue exhibited a well-organized stratified squamous epithelium overlying a dense stroma. In contrast, the organoids displayed small, spherical or cystic structures predominantly composed of epithelial cells, typically with a central eosinophilic (keratinized) core surrounded by an epithelial cell layer, and lacked the complex stromal architecture of the native tissue. The generated organoids and matched tissues were subsequently subjected to immunofluorescence staining. As shown in Figure 4B and Supplementary Figure S1, immunofluorescence staining was performed for KRT14 (epithelial), vimentin (fibroblast-associated), α-SMA (myofibroblast/smooth muscle-associated), VWF (endothelial), and SM22 (smooth muscle). In the matched tissue, KRT14 was expressed in the epithelial layer, while vimentin was observed in the stroma. In the organoids, positive expression of KRT14 was observed, with the formation of epithelial rings or clusters, whereas stromal cell-associated markers were not detected within the organoid structures, demonstrating that the vaginal organoids were composed mainly of epithelial cells. However, vimentin-positive cells were observed surrounding, but not within, KRT14-positive organoids. These cells may represent residual stromal/accessory cells. Their origin and relationship to the organoid niche require further investigation.

**FIGURE 4:**
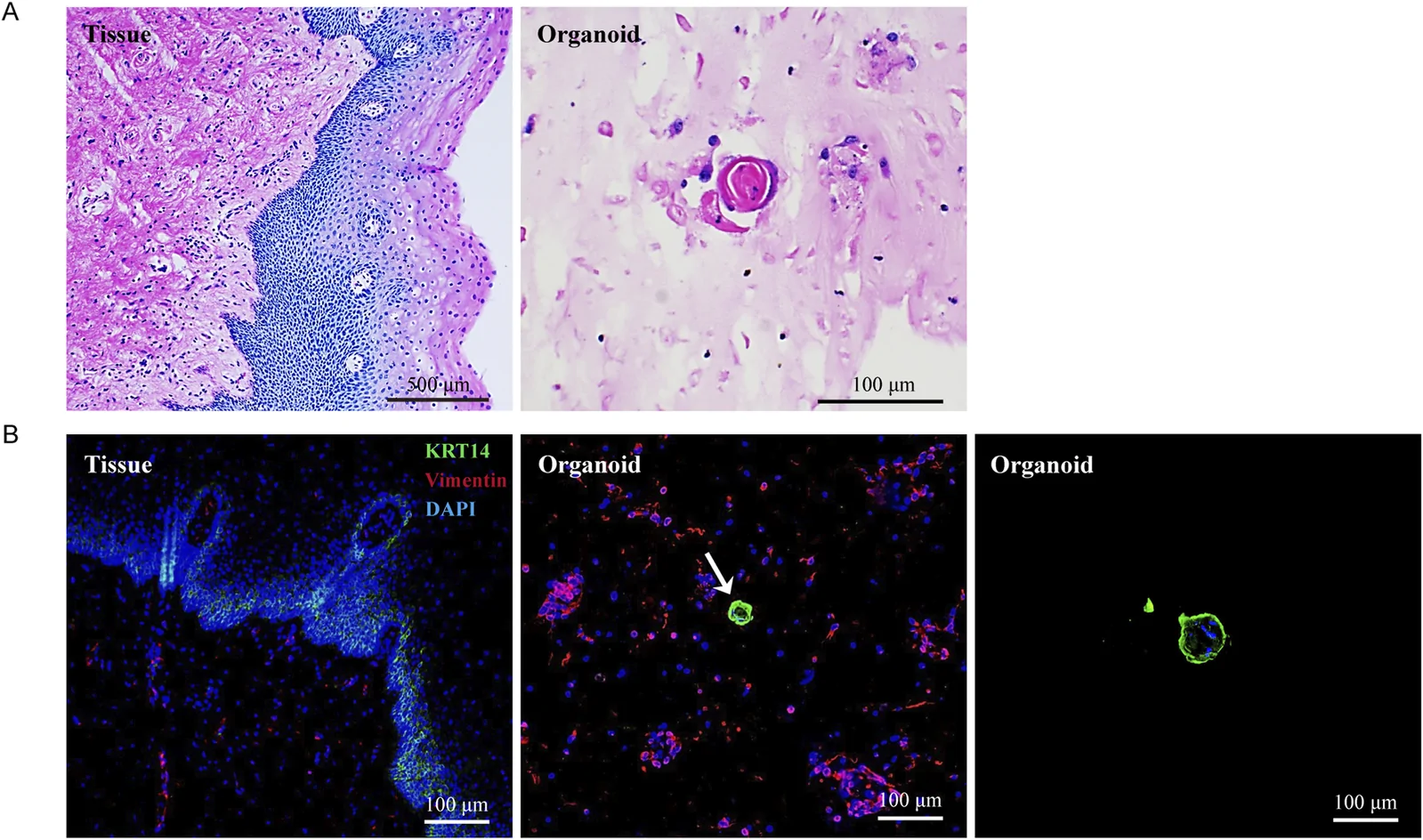
Staining of organoids and patient-matched tissues derived from the vaginal tissues of patients with POP. **(A)** H&E staining of POP vaginal tissue (left) and an organoid derived from digested tissue fragments (right). **(B)** Immunofluorescence staining for KRT14 (green), vimentin (red), and DAPI (blue) in POP vaginal tissue (left) and in organoids derived from digested tissue fragments (middle) and primary cells (right). The white arrow indicates an organoid in the middle panel. KRT14 and vimentin were used at a 1:500 dilution; DAPI was used for nuclear counterstaining. Scale bars: 500 µm (A, left) and 100 µm (A, right and B). N indicates the number of biological samples. For each biological sample, three independent technical replicates were performed.

## Discussion

This study is the first to systematically apply organoid culture technology to POP research. Using two approaches, namely, the dissociation of primary cells and the generation of digested tissue fragments, we established a reproducible protocol for generating organoids from two anatomically and functionally distinct pelvic floor tissues: the vaginal wall and the USL. Our results revealed that healthy vaginal wall and USL tissues can produce organoids, whereas tissues derived from patients with POP exhibit markedly impaired organoid-forming capacity. Our results revealed that under the conditions of organoid culture in this study, benign control vaginal wall and USL tissues were capable of producing organoids, whereas tissues derived from patients with POP exhibit markedly impaired organoid-forming capacity. To analyze the cellular composition of organoids derived from the vaginal wall, which were generated from patients with POP, we performed immunofluorescence staining; KRT14 expression may initially support epithelial cells.

Compared with that in the benign control group, the efficiency of organoid formation in the vaginal wall tissue derived from patients with POP was observed under the microscope to be lower. This difference may arise from acquired factors, such as mechanical stretch or obstetric trauma, which can lead to defects in cell renewal and tissue homeostasis (Wu et al., 2024). The reduced organoid-forming capacity of POP-derived vaginal cells, together with immunofluorescence, confirmed that these organoids may be epithelial in nature, suggesting that the number of epithelial stem/progenitor cells might have decreased or that their self-renewal and differentiation capabilities might have been impaired (Huang et al., 2020). However, additional epithelial markers should therefore be examined to confirm this interpretation. Similar phenomena have been reported in other diseases, in which pathological tissues produce fewer organoids owing to stem cell exhaustion or senescence (Vougioukalaki et al., 2022; Wang et al., 2020). Although our sample size was limited, this phenotype was consistently observed in all three patients with POP, indicating that the reduced organoid-forming capacity may reflect a generalized impairment of regenerative capacity in POP vaginal tissue and warrants further validation in larger cohorts.

Compared with the use of fully dissociated single cells, culture of digested tissue fragments increased the yield of organoids from healthy vaginal tissues, as observed under a microscope. This may be explained by the preservation of critical cell–cell and cell–matrix interactions in tissue fragments, which are disrupted during complete enzymatic dissociation in single-cell cultures (Slovackova et al., 2026). However, in POP, the extracellular matrix is known to be disorganized, with increased collagen degradation and altered proteoglycans (Tian et al., 2024; Wang et al., 2018). Therefore, the failure of tissue fragments derived from patients with POP to efficiently generate organoids may reflect not only intrinsic cellular defects but also a degraded microenvironment that can no longer support stem cell survival and self-organization.

Under the conditions of the organoid culture medium used in this study, the USL samples from the benign control group formed organoid structures, whereas the USL tissues derived from the POP source failed to produce similar structures. These findings must be interpreted cautiously; however, because the USL is a fibroblast- and smooth muscle-rich connective tissue (Ramanah et al., 2012), the medium used in our study was optimized primarily for epithelial stem cell expansion. In POP, the USL undergoes structural remodeling, including smooth muscle cell loss, increased collagen degradation, and altered fibroblast activity (Zhu et al., 2021; Bastias et al., 2025). Thus, the absence of comparable structures from POP-derived USL tissue under these conditions may reflect depletion or dysfunction of an epithelial/progenitor population, an altered stromal or smooth muscle microenvironment, or simply a mismatch between the culture system and the target cell population. Therefore, before the regenerative capacity is impaired, USL-derived cultures must be characterized using lineage-specific media or coculture systems to more precisely assess mesenchymal progenitor cell function in POP. In addition, because POP-derived USL tissue did not form organoids under the current culture conditions, immunofluorescence staining was not performed in this study. Therefore, if future studies successfully establish organoids from USL-derived cultures using lineage-specific media or coculture systems, USL-specific phenotypic markers and molecular comparisons with matched tissues will still be needed to validate the biological identity of these cultures.

Although this study established a reproducible protocol for generating organoids from the vaginal wall and the uterosacral ligament, two anatomically and functionally distinct pelvic floor tissues, several limitations remain. First, this study is limited by the very small number of patient-derived samples. Organoid formation was assessed qualitatively by microscopic observation rather than by quantitative efficiency assays. Therefore, the organoid data should be considered descriptive and preliminary, and no statistical comparison of organoid formation between groups was performed. Second, the culture system favored epithelial cell expansion, and we did not achieve stable coculture of epithelial and stromal cells within a single organoid, although coculture would more faithfully recapitulate native tissue. Finally, this study lacks molecular comparisons between the generated organoids and their matched tissues of origin. Although KRT14 expression and the absence of selected stromal, endothelial, and smooth muscle markers suggest an epithelial-enriched phenotype, these data do not establish tissue-specific identity. Therefore, future work should focus on optimizing coculture systems containing both epithelial and stromal components; generating organoids from larger cohorts with more diverse clinical characteristics to validate the preliminary differences observed here; and, importantly, quantifying organoid formation efficiency and providing statistical comparisons of this parameter across groups. In addition, qPCR, RNA-sequencing, or single-cell profiling should be used to compare control-derived and POP-derived organoids with matched native tissues and to determine whether disease-associated characteristics are retained. Ultimately, a more comprehensive understanding of the cellular and molecular mechanisms underlying POP will be essential for developing nonsurgical therapeutic strategies and improving long-term treatment outcomes for patients with POP.

## Data Availability

The original contributions presented in the study are included in the article/Supplementary Material, further inquiries can be directed to the corresponding authors.
