# Supplementary material for: A protocol for vaginal wall-derived organoids from women with pelvic organ prolapse and benign controls: limited applicability to pathological uterosacral ligament tissues
Source: Front Cell Dev Biol. 2026 Oct 23;14:1977193. doi: 10.3389/fcell.2026.1977193 (PMC13739802; doi:10.3389/fcell.2026.1977193)
Supplement: Supplementary file 1 [file Supplementaryfile1.docx]

Supplementary Material

# 1 Supplementary Methods

# Detailed step-by-step organoid generation protocol

This section contains the complete procedural workflow corresponding to the condensed Methods in the main text.

Tissue collection and transportation (Steps 1–3)

(1)	Precool the tissue storage solution at 4 °C, and take it to operating room in crushed ice for tissue collection.

(2)	Rinse the tissue with saline firstly to remove blood, then cut it to 2-3 cm^3^ size and transfer to a 15-mL conical tube containing 10-12 mL of ice-cold tissue storage solution.

(3)	Keep tissues in storage solution at 4 °C and transport to laboratory as soon as possible (≤48 h, ideally <2 h).

Tissue pretreatment and digestion (Steps 4–11)

(4)	Take out sample tissue to a 6-well plate on the ice, and wash it with 3 mL pre-cool wash solution.

(5)	Transfer it to a 15-mL Falcon tube with 12 mL wash solution for further wash by shaking the tube. Take out sample tissue to a new 15- mL Falcon tube, and add new wash solution. Repeat such wash for 3 times.

(6)	Put the washed tissue in a 6-well plate on the ice, and use surgical scissors to mince the tissue into fragments of 5 mm^3^.

(7)	Fix some representative fragments in 4% paraformaldehyde for further histological analysis.

(8)	Remained tissue fragments are transferred to a 1.5-mL Eppendorf tube, and add 0.3 mL tissue digestion solution, then use surgical scissors to cut them into small fragments of 1-3 mm^3^.

(9)	Add 0.7 mL tissue digestion solution to the1.5-mL Eppendorf tube, and transfer the tissue fragments to a new 15-mL Falcon tube.

(10)	Place the Falcon tube containing 10 mL of digestion solution and tissue fragments on a thermostatic oscillator. Digest the tissue with a moderate oscillation frequency at 90-100 rpm for 1 h under 37 °C.

(11)	Transfer digestion mixture to a 50-mL tube，and add 10 mL of DMEM containing 10% FBS to stop the enzymatic reaction.

Primary cells plating (Steps 12–25)

(12)	Filter the final mixture through a 100-µm cell strainer.

(13)	Collect filtered cells and centrifuge at 400 g for 5 min.

(14)	Remove the supernatant, then resuspend in 10 mL of wash buffer.

Note: All the steps of wash and filtration, the tubes are kept on ice to maintain the cell viability.

(15)	Aspirate wash solution, then resuspend in 1 mL ACK lysis buffer at room temperature, incubating for 2-3 min.

(16)	Dilute the lysis with 3 mL of DMEM, then centrifuge at 400 g for 5 min.

(17)	Remove the supernatant, and resuspend the pellet in 0.5 mL of wash buffer. Aspirate 25 µL of cell suspension to mix with 25 µL of trypan blue for cell counting and viability detection.

Note: The sample with higher ratio of living cells is more likely to generate organoids. In this study, the sample with a proportion of living cells more than 50% are used.

(18)	Centrifuge remained cell suspension at 400 g for 5 min.

(19)	Remove supernatant, then resuspend the pellet in Matrigel. Approximately 50,000 cells are plated in 35 µL of Matrigel.

Critical: The usage of Matrigel must be conducted on ice to prevent its solidification.

Note: To avoid attachment of Matrigel to pipette tip, the pipette tip is washed with anti-adherence rinsing solution before using. The dilution of Matrigel should be less than 20%, otherwise the formed solid droplets may dissolve during organoid culture.

Note: When mixing Matrigel and cells, avoid introducing bubbles, which may also influence droplets stability and interfere following imaging. If there are bubbles in mixture, leaving the tube on ice for several minutes can be beneficial for their disappearance.

(20)	Plate the mixture of cells and Matrigel on a pre-heated 24-well plate with 35 µL of one drop.

Critical: When plating cells, work as quickly as possible to prevent Matrigel from solidifying in pipette tips.

Note: The plate is preheated in cell incubator at 37 °C before use. It helps to accelerate Matrigel drops solidification, bringing stable 3D structure and decreasing cell attachment to the bottom of plate, beneficial for generating organoids.

Note: The large volume of each drop can influence its structural stability and culture medium penetration. Small drops are advised for plating. If there are a large amount of Matrigel, the 12-well or 6-well plates can be utilized. Among them, 3~4 drops of Matrigel are plated in a 12-well plate, and 10~12 drops of Matrigel are plated in a 6-well plate.

(21)	Place the plate to the cell incubator for 20 min at 37 °C, 5% CO2.

(22)	After drops solidification, carefully add 700 µL of organoid culture medium to each well.

Critical: Avoid directly adding the medium on the top of Matrigel drops, as it may disrupt them.

(23)	Add 10 µM ROCK inhibitor Y-27632 in the medium of each well, and incubate the plate at 37 °C and 5% CO2.

(24)	After 2 days, change fresh culture medium without Y-27632.

(25)	Replace the culture medium without Y-27632 every 3 days.

Note: When removing the medium, hold the plate at a 45° angle and work slowly, avoiding pipette tips breaking Matrigel drops.

Note: The example of organoid growth is observed in Figure 1and Figure 3.

Organoid generation from digested tissue fragments (Steps 26–34, optional)

In Step 12, the retained tissue fragments on the cell strainer can also be collected for organoid generation.

(26)	Use pipettes with a cut-off tip to transfer retained tissue fragments from the cell strainer to a 15-mL Falcon tube.

(27)	Add 5 mL of DMEM, then centrifuge at 400 g for 5 min. Centrifuge at 400 g for 5 min.

(28)	Remove supernatant, then resuspend the pellet in 5 mL of DMEM again.

(29)	Remove supernatant, then resuspend the pellet in 3 mL of organoid culture medium containing 50% Matrigel.

(30)	Transfer the tissue suspension to a 6-well low-attachment plate.

Note: Low-attachment plate can reduce the adhesion growth of cells.

(31)	Add 10 µM ROCK inhibitor Y-27632 in the medium of well, and incubate the plate at 37 °C, 5% CO2.

(32)	After 2 days, collect the culture medium and tissue fragments to a 15-mL Falcon tube, then centrifuge at 400 g for 5 min.

(33)	Remove supernatant and add fresh culture medium containing 50% Matrigel without Y-27632.

(34)	Replace the culture medium containing 50% Matrigel without Y-27632 every 2 days.

Organoid passaging (Steps 35–41)

(35)	Passage when cell aggregate/organoid size is among 100-300 µm.

(36)	Collect the culture medium and tissue fragments to a 15-mL Falcon tube, pipette up and down, then centrifuge at 400 g for 5 min. Aspirate supernatant, add 1mL TrypLE and incubate at 37 °C for about 10 min.

Note: During incubation, every 3 min pipette up and down with a P1000 pipette tip about 10 times every 3 min to accelerate disruption of cell aggregates/organoids.

Critical: Closely monitor organoid dissociation with microscope. When there are small cell clumps (consisting of 5-20 cells), digestion is finished. Digestion time do not exceed 15 min. Too long dissociation may reduce organoid outgrowth.

(37)	Dilute TrypLE with 5 mL of DMEM containing 2% FBS. Centrifuge at 400 g for 5 min.

(38)	Remove supernatant and resuspend the pellet in 2 mL of PBS. Dye with Trypan blue at a ratio of 1:1 for cell counting.

(39)	Centrifuge at 400 g for 5 min. Then resuspend the pellet in Matrigel (~50000 cells/35 µL of Matrigel).

(40)	Plate the cell suspension on pre-heated 24-well plate, 35 µL of one drop into each well. Incubate the plate at 37 °C and 5% CO2 for 20 min to solidify Matrigel.

(41)	Carefully add 700 µL of organoid medium to each well, and every 3 days change fresh culture medium.

Note: The example of organoid generation from digested tissue fragments is observed in Figure 2.

Organoid recovery and fixation (Steps 42–48)

(42)	Remove organoid culture medium, and wash with pre-cool HBSS for twice in the well.

(43)	Add 1 mL of pre-cool Cell Recovery Solution to each well, and pipette up and down with P200 pipette tip adequately on ice.

(44)	Place the plate to a shaker at 4 °C for moderate shaking 15-30 min to promote Matrigel degradation.

(45)	Aspirate the to a 15-mL Falcon tube, mix with 5 mL of pre-cool HBSS, and centrifuge at 400 g for 5 min.

Note: After centrifugation, mostly of organoids deposit at the bottom of the tube, thus organoids have good recovery. If many organoids are still trapped in Matrigel, repeat the Step 42-45, and make a proper increase of centrifugal force to 500~600 g.

(46)	Remove supernatant and wash the pellet with 2 mL of pre-cool HBSS.

(47)	Centrifuge at 400 g for 5 min.

(48)	Remove supernatant, then resuspend the pellet in 1 mL of pre-cool 4% paraformaldehyde for organoids fixation, transferring the mixture to a 1.5-mL Eppendorf tube.

Note: At room temperature, the fixation time is 2 h. If there are no experiment plans, the fixed organoids can be stored at 4 °C.

Immunofluorescence staining (Steps 49–65)

(49)	Centrifuge at 400 g for 5 min. Remove fixation solution, and wash the pellet with 1 mL of pre-cool HBSS.

(50)	Centrifuge at 400 g for 5 min, repeat the wash step twice.

(51)	Remove supernatant, then pre-embed the organoids with agarose solution, and place the pre-embedded organoids at 4 °C overnight for agarose solidify.

(52)	Take pre-embedded organoids to 30% sucrose solution at 4 °C overnight for dehydration.

(53)	Use OCT agent to further embed the organoids, then cut them to 8 µm-slices with freezing microtome.

Note: The obtained slices and OCT-embedding organoids should be stored at -80 °C.

(54)	Before immunofluorescent staining, put the cold slices at room temperature for 10 min.

(55)	Wash the slices with PBS solution twice, each time for 5 min.

(56)	Permeabilize the slices with 0.5% Triton X-100 solution for 30 min at room temperature.

(57)	Wash the slices with PBS solution twice.

(58)	Block the slices with 3% BSA solution for 1 h at room temperature.

(59)	Cover the slices with diluted primary antibody and incubate at 4 °C overnight.

Note: The incubation time usually is about 16~24 h, but it can be extended to 48 h if needed.

(60)	Wash the slices with PBS solution five times.

Note: Adequate wash is beneficial for reducing false positive results.

(61)	Cover the slices with diluted secondary antibody and incubate at room temperature for 2 h.

Note: Place away from light.

(62)	Wash the slices with PBS solution three times.

(63)	Cover the slices with dapi at room temperature for 10 min.

(64)	Wash the slices with PBS solution three times.

(65)	Add 40 µL of antifade mounting medium to the slices and then carefully put the cover glasses.

Note: Avoid introducing bubbles. Keep the slices at 4 °C and away from light. Image the slices as early as possible.

Note: The example of organoid immunofluorescent staining is observed in Figure 4.

# 2 Supplementary Figures and Tables

**Table S1.** Composition of reagents

| Reagent | Components | Storage |
| --- | --- | --- |
| Tissue storage solution | adDMEM/F12 + 1% P/S + 10 mM HEPES + 25 µg/mL gentamicin + 1% GlutaMAX + 10 µM Y-27632 + 1% FBS | 4°C (use within 1 month; -20°C for long-term) |
| Wash solution | PBS + 5% P/S | 4°C, prepare fresh |
| Digestion solution | DMEM + 2.5 mg/mL Collagenase II + 0.5 mg/mL DNase I | -20°C (≤1 month) |
| Digestion arrest solution | DMEM + 10% FBS | 4°C, prepare fresh |
| Organoid culture medium | adDMEM/F12 + 1% GlutaMAX + 10 mM HEPES + 1% P/S + 25 µg/mL gentamicin + 2.5 mM nicotinamide + 1.25 mM N-acetyl-L-cysteine + 1× B-27 + 200 ng/mL Wnt3a + 200 ng/mL R-Spondin 1 + 50 ng/mL Noggin + 100 ng/mL FGF10 + 50 ng/mL FGF7 + 10 µM forskolin + 0.5 µM A83-01 + 1 µM SB202190 + 1 µM CHIR-99021 | 4°C, use within 2 weeks |

**Table S2.** Composition of reagents

| **Name** | **Company** | **Catalog number** |
| --- | --- | --- |
| **Equipment** |  |  |
| Cell culture 6-well plate | Nest Biotech | 703001 |
| Cell culture 24-well plate | Nest Biotech | 712001 |
| Cell strainer 100 μm | Biosharp | BS-100-XBS |
| Surgical scissor | Shinva | ZC343R |
| Stainless-steel forceps | VETUS | ST-10 |
| 15-mL conical tube | Bioland | ATS05-15 |
| 50-mL conical tube | Bioland | ATS05-50 |
| 1.5-mL Eppendorf tube | QSP | 509-GRD-Q |
| Tips, p1000 | QSP | 112NXL-Q |
| Tips, p200 | QSP | T090-Q |
| 6-well low-attachment plate | Corning | 3471 |
| **Reagents** |  |  |
| Advanced DMEM/F12 (adDMEM/F12) | Gibco | 12634010 |
| Penicillin-streptomycin (P/S) | Gibco | 15140-122 |
| HEPES, Free Acid,Cell Culture Grade | Yeasen | 60110ES76 |
| Gentamycin | Yeasen | 60214ES03 |
| GlutaMAX supplement | Gibco | 35050061 |
| Y-27632 | Yeasen | 53006ES10 |
| Fetal bovine serum (FBS) | Gibco | A5669701 |
| DMEM | Gibco | C11995500BT |
| Collagenase II | Sigma | V900892-1G |
| Nicotinamide | Yeasen | 51402ES08 |
| N-acetyl-L-cysteine | Yeasen | 50303ES05 |
| B-27 supplement | Gibco | 17504044 |
| Recombinant human Wnt-3a protein | Novoprotein | C22R |
| Recombinant human R-Spondin 1 protein | Sino Biological | 11083-HNAS |
| Recombinant human Noggin protein | Sino Biological | 10267-HNAH |
| Recombinant human fibroblast growth factor 10 (FGF10) | Sino Biological | 10573-HNAE |
| Recombinant human fibroblast growth factor 7 (FGF7) | Sino Biological | 10210-H07E |
| Forskolin | MCE | HY-15371 |
| A83-01 | MCE | HY-10432 |
| SB202190 | MCE | HY-10295 |
| CHIR-99021 | MCE | HY-10182 |
| PBS buffer | Biosharp | BL601A |
| 4% paraformaldehyde | Beyotime | P0099-100mL |
| Trypan blue solution | Solarbio | C0040 |
| ACK lysing buffer | Solarbio | R1010 |
| Matrigel | Corning | 356231 |
| TrypLE | Gibco | 12604021 |
| Cell Recovery Solution | Corning | 354253 |
| OCT mounting media | SAKURA | 4583 |
| HBSS, w/o calcium and magnesium | Solarbio | H1040 |
| Sucrose | Aladdin | S112228-500g |
| Bovine Serum Albumin (BSA) | Aladdin | A104912-25g |
| Triton X-100 | Aladdin | 232-658-1 |
| DAPI | Solarbio | C0065 |
| Alpha smooth muscle actin (α-SMA) | Abcam | ab32575 |
| Smooth muscle protein 22-alpha (SM22) | Abcam | ab10135 |
| Cytokeratin 14 (KRT14) | Abcam | ab7800 |
| Von willebrand factor (VWF) | Abcam | ab201336 |
| Vimentin | Abcam | ab92547 |
| DyLight 488 AffiniPure-conjugated secondary antibodies | EarthOx | E032211 |
| DyLight 549 AffiniPure-conjugated secondary antibodies | EarthOx | E032310 |

**Table S3. Troubleshooting and solutions.**

| **Step** | **Problem** |  | **Possible reason** |  | **Solution** |
| --- | --- | --- | --- | --- | --- |
| 20 | Matrigel drops dispersion |  | Drops are too big, or Matrigel are diluted too much |  | 1) Keep the drops in small size (recommend 10∽40 μL/drop); 2) before adding Matrigel to the cell pellet, the supernatant must be removed as clear as possible; 3) pre-heat the plate more than 30 min. |
| 22 | Matrigel drops collapse |  | The operation is rough during transferring the plate to incubator |  | No matter for taking the plate or placing it to the incubator, operate carefully as well as fast |
| 24-25 | No organoid generation |  | Low cell viability or low proportion of epithelial cells |  | 1) Put the tissue to storage solution as soon as possible after cutting up; 2) shorten transport time and conduct the experiment as early as possible; 3) throughout the whole generation process, avoid tissue or cells exposure to air; 4) choose suitable tissue sample. |
| 32 | No cell aggregates generation |  | Low cell viability or tissues are fully digested |  | 1) Choose suitable tissue sample and conduct experiment quickly; 2) shorten digestion time. |
| 36-41 | Organoids loss |  | Low organoids recovery or cell adhere on the tip |  | 1) Wash tips with anti-adherence rinsing solution before using; 2) the mixture of organoids and Matrigel is put on the ice for 5-10 min before centrifuging or plating |
| 53 | Organoids slices are easily cracked |  | Organoids are not dehydrated enough |  | Extended dehydration time. Generally when the organoids naturally settles to the bottom of the tube suggests dehydration finish |

**Figure S1.** Immunofluorescent staining of α-SMA, VWF and SM22 in vaginal tissue of pelvic organ prolapse patient
